# Utility of fetal cardiovascular magnetic resonance imaging in assessing the fetuses with complete vascular ring

**DOI:** 10.3389/fped.2023.1159130

**Published:** 2023-04-11

**Authors:** Xia Zhang, Ming Zhu, Su-Zhen Dong

**Affiliations:** Department of Radiology, Shanghai Children’s Medical Center, School of Medicine, Shanghai Jiao Tong University, Shanghai, China

**Keywords:** fetal heart, magnetic resonance imaging, aortic arch, ductus arteriosus, congenital heart disease, prenatal diagnosis

## Abstract

**Objective:**

This study aimed to report our experience in qualitative and quantitative evaluation of fetal complete vascular ring (CVR) using fetal cardiovascular magnetic resonance imaging (MRI) to improve prenatal diagnosis and make early postnatal management possible.

**Methods:**

A retrospective case-control study was performed on cases of CVR diagnosed using fetal cardiovascular MRI, and confirmed by postnatal imaging diagnosis. Associated abnormalities were recorded. The diameters of aortic arch isthmus (AoI) and ductus arteriosus (DA), and tracheal diameters in fetuses with tracheal compression were measured and compared with those of the control group.

**Results:**

All fetal CVR cases in this study included right aortic arch (RAA) with aberrant left subclavian artery (ALSA) and left DA (*n* = 93), double aortic arch (DAA) (*n* = 29), RAA with mirror-image branching and retroesophageal left ductus arteriosus (RLDA) (*n* = 8). Compared with the control group, the diameters of AoI in fetuses with DAA were decreased (*p* < 0.001), and the diameters of DA in fetuses with RAA with ALSA and left DA were increased (*p* < 0.001). The diameters of AoI and DA were positively correlated with gestational age (GA) in the normal control group (both *p* < 0.001); The diameters of AoI and DA were also positively correlated with GA in RAA with ALSA and left DA subgroup (both *p* < 0.001) and RAA with mirror-image branching and RLDA subgroup (AoI: *p* = 0.003; DA: *p* = 0.002); The diameters of DA were positively associated with GA in DAA subgroup (*p* < 0.001), however, there was no linear tendency between the diameters of AoI and GA in the DAA subgroup (*p* = 0.074). There were CVR fetuses with associated intracardiac malformation (*n* = 13), especially ventricular septal defect rather than complex heart disease, and extracardiac malformation (*n* = 14). Sixteen fetuses were shown the airway compression whose tracheal diameters were smaller than the normal (*p* < 0.001).

**Conclusions:**

The altered diameters of AoI and DA can be detected and measured in CVR fetuses using fetal cardiovascular MRI. Fetal CVR can occur alone or with intracardiac and extracardiac malformation. Fetal CVR can be associated with prenatal airway compression.

## 1. Introduction

Vascular ring is a congenital anomaly of the aortic arch (AA) that occurs in only 1/10,000 live births (1). During fetal development, a series of arches regress to form AA. When the arches regress in an abnormal pattern, AA and its branches appear around the esophagus and trachea, rather than in front of them, they form a vascular ring. Based on the theoretical embryopathogenesis, the International Congenital Heart Surgery Nomenclature and Database Committee classified vascular rings as the complete vascular ring (CVR) and incomplete vascular ring (2). The CVRs include right aortic arch (RAA) with aberrant left subclavian artery (ALSA) and left ductus arteriosus (DA), double aortic arch (DAA), right aortic arch (RAA) with mirror-image branching and retroesophageal left ductus arteriosus (RLDA); The incomplete vascular rings include innominate artery compression syndrome, pulmonary artery sling, left aortic arch with aberrant right subclavian artery (2, 3). In CVR, trachea and esophagus are completely encircled by a composite vascular structure, which is formed by the abnormal AA, ductus arteriosus (DA) (3). Postnatal CVR may be asymptomatic or cause respiratory and/or gastrointestinal symptoms. The severity of symptoms and the time of onset of CVR mainly depend on the diameters and positions of AA and its branches, DA, trachea, and associated intracardiac/extracardiac malformations (4). A thorough understanding of embryonic development of AA and its branching pattern variants can help to understand and classify CVR, which further contributes to risk stratification, providing more informed family counseling and providing opportunities for early intervention.

The current standard diagnosis method for fetal CVR is fetal echocardiography with a three-vessel view. However, echography has some limitations that impair visualization of fetal cardiovascular structures including maternal obesity, fetal position, uterine fibroids, twins and oligohydramnios. Previous studies have shown that fetal cardiovascular MRI was a useful adjunct to ultrasound when ultrasound was technically limited (5). However, so far, there are few studies reported on the qualitative evaluation of CVR using fetal cardiovascular MRI, the few published studies were only qualitative diagnostic studies with a small sample of DAA and RAA (6, 7). To the best of our knowledge, no case of CVR quantitatively assessed by fetal cardiovascular MRI has been reported. Thus, the purpose of this retrospective study was to report our experience with the diagnosis fetal CVR based on fetal AA and its branching pattern variants using fetal cardiovascular MRI, and further compare the positions and diameters of aortic arch isthmus (AoI) and DA in fetuses between different subtypes of CVR and those of normal control group to improve prenatal diagnosis and make early postnatal management possible.

## 2. Methods

### 2.1. Subjects

This study was approved by the ethics committee of Shanghai Children's Medical Center (Approval number: SCMCIRB-K2016001). The written consent was obtained from all pregnant mothers.

**Figure 1:**
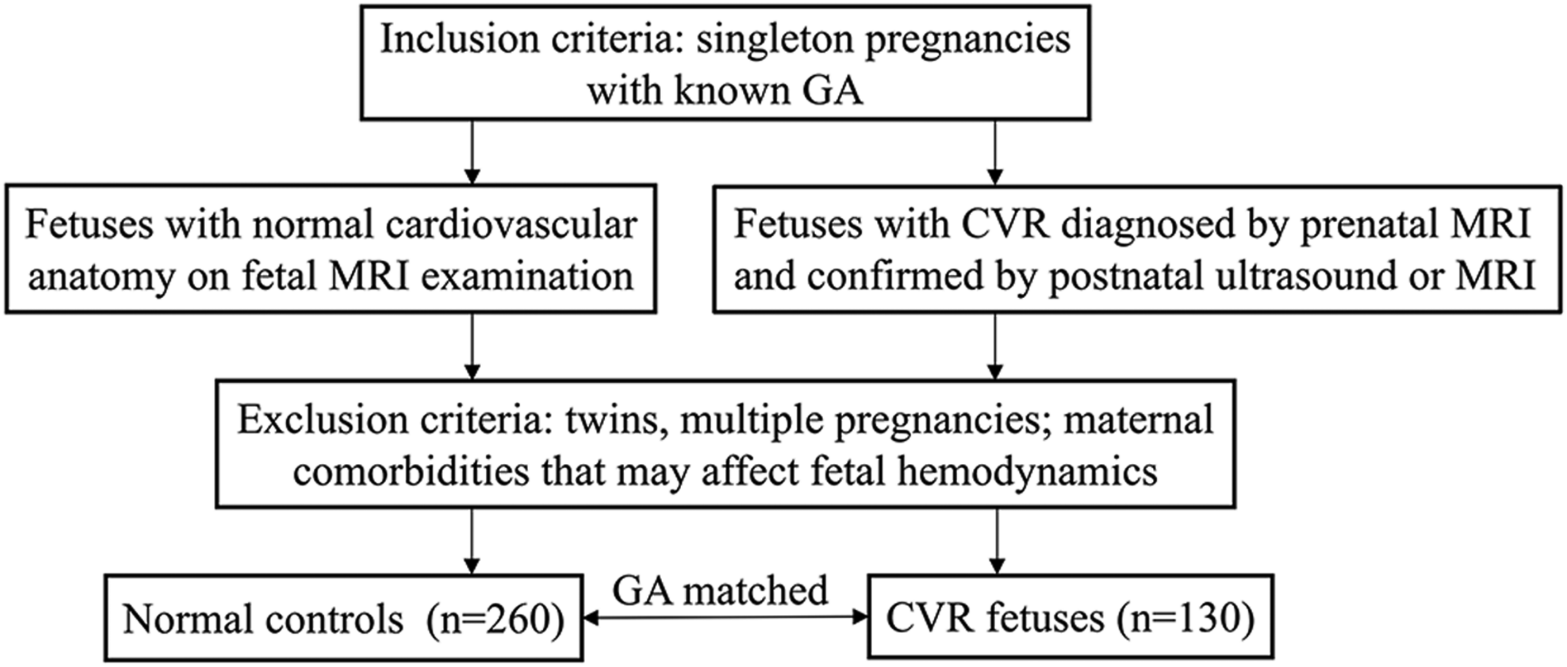
Flowchart showing the subjects inclusion and exclusion criteria. GA, gestational age; CVR, complete vascular ring; MRI, magnetic resonance imaging.

This was a retrospective case-control study performed from September 2013 to June 2022. Inclusion criteria: singleton pregnancies with known gestational age (GA). Fetuses with normal cardiovascular anatomy on fetal MRI were enrolled in the normal control group when ultrasound was technically limited. Fetuses with CVR diagnosed by prenatal MRI and confirmed by postnatal ultrasound or MRI were included in the CVR groups. No subjects prenatally diagnosed with CVR but not confirmed by postnatal ultrasound or MRI. CVR group and normal control group fetuses were matched based on GA. Exclusion criteria: twins, multiple pregnancies; maternal comorbidities that may affect fetal hemodynamics (Figure 1).

### 2.2. Fetal cardiovascular MRI

Fetal cardiovascular MRI was performed using a 1.5 T unit (Philips Medical Systems, BEST, The Netherlands) with 60 mT/m gradients and a 16-channel sense-xl-torso coil. Fetal cardiovascular MRI scan sequences included steady-state free precession (SSFP), single-shot turbo spin echo (SSTSE), real-time SSFP cine, and non-gated phase contrast (PC) sequences. SSFP sequence was used for assessment of fetal cardiovascular structure; SSTSE sequence was used for showing the bronchus and assessment of the visceroatrial situs; Real-time SSFP cine sequence was used for showing the beating heart. Non-gated PC sequence was used for showing blood flow direction of the vessels. The combination of SSFP sequence with the radial k-space sampling technique was adopted to decrease the number of artefacts. No sedation and cardiac gating were used. The total acquisition time was 20–30 min. Detailed imaging parameters are summarized in Table 1.

**Table 1:** Detailed parameters of fetal cardiovascular MRI sequences.

	TR (ms)	TE (ms)	FOV (mm^2^)	Matrix	Slice thickness (mm)	Spacing (mm)	Flip angle
SSFP	3.6	1.8	260–325	216 × 218	2–4	−2–0	70°–80°
SSTSE	12,000	80	260–355	236 × 220	2	0	90°
Real-time SSFP cine	2.7	1.34	280–310	128 × 128	8	−6	65°
PC	7.9	4.8	300	232 × 230	5	0	12°

SSFP, steady-state free-precession; SSTSE, single-shot turbo spin echo; PC, phase contrast; TR, Repetition Time, TE, Echo Time; FOV, field of view.

**Figure 2:**
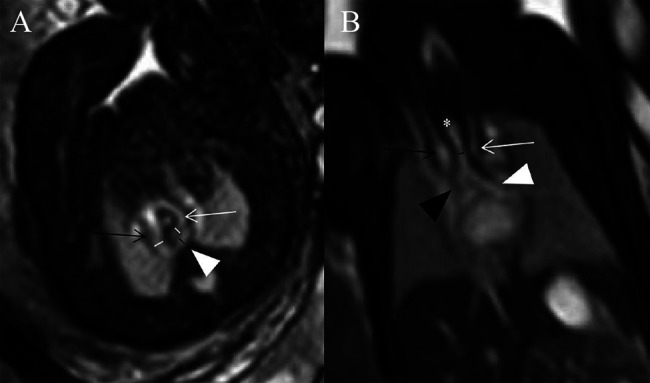
(**A,B**) Diagrams of measuring the diameters of AoI, DA, and tracheal in a 29 weeks’ gestation fetus with DAA. (**A**) Measurements of AoI and DA diameter in the transverse view of AA. Fetal cardiovascular magnetic resonance SSFP sequence transverse view of AA image showed AoI (white lines) and DA (black line) diameter measured, right aortic arch (black open arrow), left aortic arch (white open arrow), left-sided DA (white arrowhead); (**B**) measurement of tracheal diameter on the coronal view. Fetal cardiovascular magnetic resonance SSFP sequence coronal view image showed the trachea (white asterisk), tracheal diameter measured (black line), right aortic arch (black open arrow), left aortic arch (white open arrow), right principal bronchus (black arrowhead), left principal bronchus (white arrowhead).

A structural anatomical assessment on fetal MRI was carried out retrospectively by two senior radiologists with 18 and 30 years of experience in pediatric MRI respectively (S-ZD and MZ). Fetal cardiovascular structures were analyzed using a modified anatomic segmental approach for CHD reported previously (5). The assessment of fetal cardiovascular structure mainly included the visceroatrial situs, position of the heart, position of the inferior vena cava and aorta relative to the midline, ventricular looping, ventriculo-arterial connections, aortic arch with branching, ductus arteriosus, trachea and esophagus, systemic and pulmonary venous connections, and intracardiac structures. A single pediatric radiologist with 3 years of experience in fetal cardiovascular MRI (XZ) measured the diameters of AoI and DA in all cases. The diameters of the AoI and DA were measured in the transverse view of AA before they entered into the descending aorta on SSFP imaging (8) (Figure 2A). All measurements were shown as the average of the two measurements. Congenital heart defects (CHDs) and extracardiac malformation and tracheal compression of all fetuses were recorded. The trachea was estimated from repetitive observations of the axial, coronal and sagittal planes. The diagnoses of tracheal compression were made based on a consensus between two radiologists (S-ZD and MZ). The tracheal diameters of fetuses with tracheal compression were measured in the coronal SSFP sequences views (showing the best configuration of the trachea) by another pediatric radiologist (XZ) (9) (Figure 2B).

As for the measurements of AoI and DA diameters, a subgroup of 30 randomly selected fetuses was re-analyzed by the same operator (XZ) after a 2-month interval to investigate intra-observer reproducibility. 30 randomly selected fetuses were also analyzed by two independent MRI observers (S-ZD and MZ) to study inter-observer reliability. As for the measurements of tracheal diameters, intra-observer's agreement was calculated by comparing the measured value obtained twice with a 2-month interval by the same observer (XZ). Inter-observer agreements were investigated by two MRI observers (S-ZD and MZ). They measured 16 tracheal inner diameters of fetuses with tracheal compression independently and were blinded to each other's measurements.

### 2.3. Statistical analysis

Spss 26.0 software was used to analyse data. The mean, standard deviation (SD), minimum, maximum, median, and 25th and 75th percentile for AoI and DA diameters were calculated in control and three CVR subgroups. Tracheal diameters were presented as the median (25th–75th percentiles) in fetuses with tracheal compression. Inter-and intra-observer variability was calculated using the intraclass correlation coefficient (ICC). Q: Q plots were used to confirm a normal distribution of values. Data conforming to normal distribution were analyzed using an independent sample *t-*test, and non-normal distribution data were analyzed using Mann–Whitney *U*-test and Spearman analysis. The diameters of AoI and DA between normal group and three CVR subgroups were compared using independent sample *t-*test and Mann–Whitney *U*-test. In reference to the published measurements of tracheal diameters in normal fetuses (10), Mann–Whitney *U*-test was to detect difference between fetuses with tracheal compression and GA-matched normal fetuses. The Pearson analysis and the Spearman analysis were used to evaluate the correlations between vascular diameters of AoI and DA and GA. *P* < 0.05 was considered statistically significant.

## 3. Results

**Figure 3:**
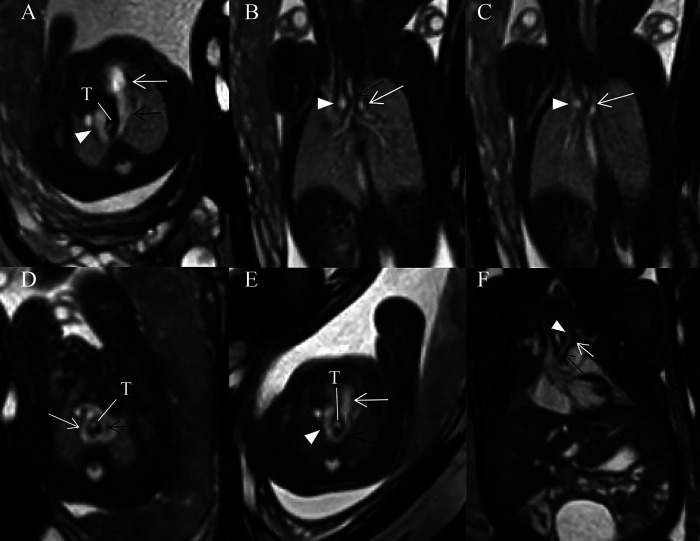
(**A–F**) Fetuses with subtype CVRs. (**A**) A 27 weeks’ gestation fetus with RAA with ALSA and left DA. Fetal cardiovascular magnetic resonance SSFP sequence transverse view of AA image showed right aortic arch (white arrowhead), left-sided DA (black open arrow) and main pulmonary artery (white open arrow) forming a U-shaped loop around the fetal trachea (T). (**B**) Same fetus with (**A**). Fetal cardiovascular magnetic resonance SSFP sequence coronary view image showed the trachea stenosis (black open arrow), right aortic arch (white arrowhead) and aberrant left subclavian artery (white open arrow). (**C**) Same fetus as (**A**). Fetal cardiovascular magnetic resonance SSFP sequence coronary view image showed right aortic arch (white arrowhead) and aberrant left subclavian artery (white open arrow). (**D**) A 28 weeks’ gestation fetus with DAA. Fetal cardiovascular magnetic resonance SSFP sequence transverse view of AA image showed right (white open arrow, larger) and left aortic arches (black open arrow, smaller) forming an O-shaped loop around the trachea (T). (**E**) A 25 weeks’ gestation fetus with RAA with mirror-image branching and RLDA. Fetal cardiovascular magnetic resonance SSFP sequence transverse view of AA image showed retroesophageal ductus (black open arrow), right aortic arch (white arrowhead), and main pulmonary artery (white open arrow). A U-shaped loop around the fetal trachea (T). (**F**) A 37 weeks’ gestation fetus with RAA with mirror-image branching and RLDA. Fetal cardiac magnetic resonance SSFP coronal view image showed left innominate artery (black arrow), left common carotid artery (white arrowhead), and left subclavian artery (white open arrow).

This retrospective study included 390 pregnant women (130 carrying fetuses with CVR, 260 carrying healthy fetuses). The GA of these fetuses at time of fetal MRI ranged from 22 to 37 weeks (mean, 26.3 weeks) both in normal control and CVR groups. Fetal CVR included RAA with ALSA and left DA (Figures 3A–C) (*n* = 93), DAA (Figure 3D) (*n* = 29), and RAA with mirror-image branching and RLDA (Figures 3E,F) (*n* = 8). 29 confirmed cases of DAA included 19 cases of right dominant arch type, 5 cases of balanced arch type and 5 cases of left dominant arch type. Except for one case of bilateral DA, the remaining DAA fetuses were all with left-sided DA.

As for MRI diagnosis of fetal vascular rings, when a U-shaped vascular loop was formed around the trachea, with persistent RAA, left descending aorta, and left DA, with persistent RAA, left descending aorta, and left DA, RAA with ALSA and left DA was diagnosed; when the ascending aorta was divided into the right and left AAs surrounding the trachea and esophagus with an O-shape and converge into the descending aorta in the transverse AA view, DAA was diagnosed; when a U-shaped vascular loop was formed with left DA extending from left pulmonary artery through the posterior side of the esophagus to left proximal descending aorta in the transverse view, but there is no aberrant left subclavian artery in the coronal view, RAA with mirror-image branching and RLDA was diagnosed.

**Figure 4:**
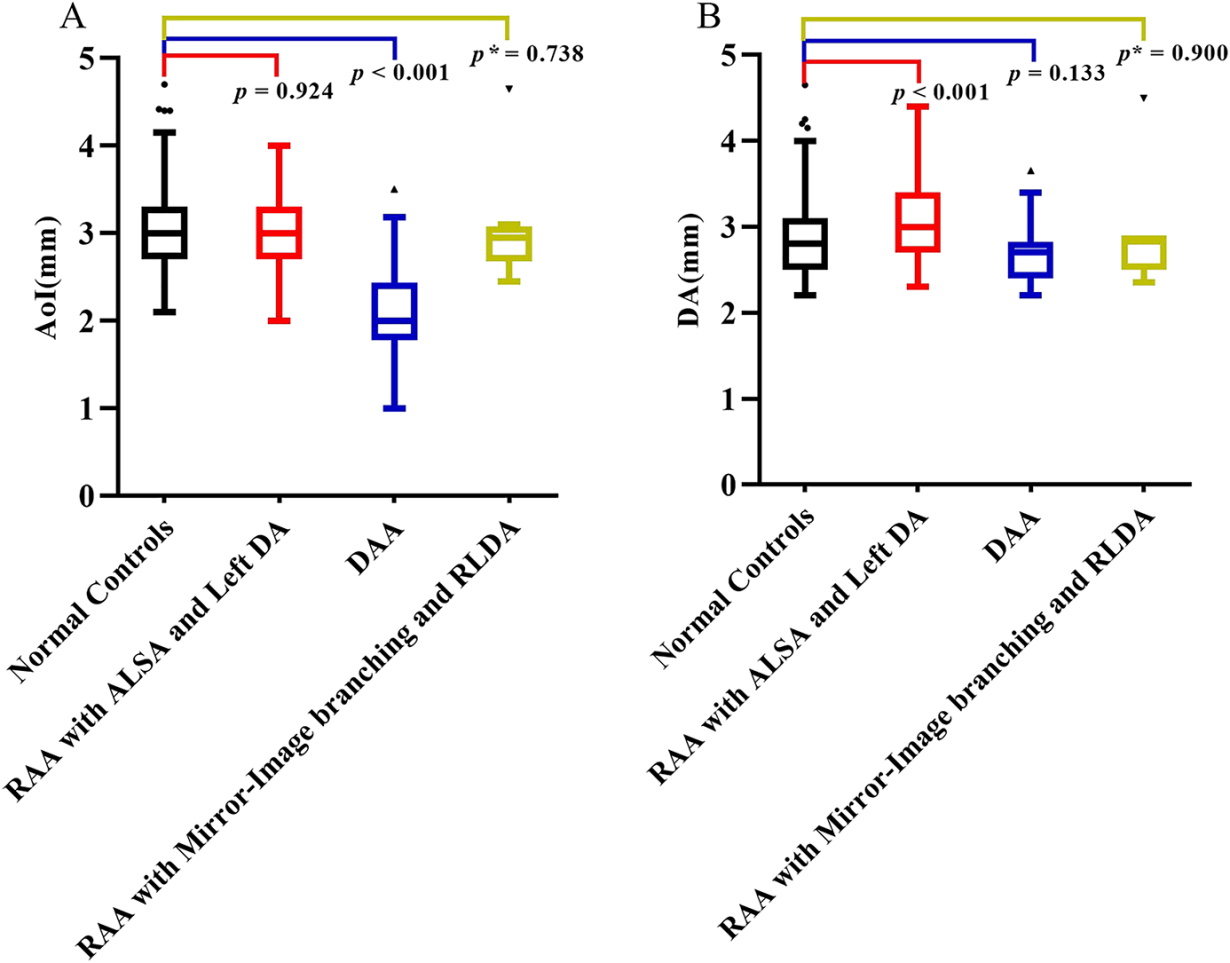
(**A,B**) Comparison of AoI and DA diameters measured by fetal MRI. (**A**) Box-graph showed the differences of the diameters of AoI between the normal controls group and three subgroups of CVR; (**B**) box-graph showed the differences of diameters of DA between normal controls group and three subgroups of CVR. *p* < 0.05 was considered statistically significant. *p* refers for independent sample *t*-test, and p* refers for Mann-Whitney *U*-test. AoI, aortic arch isthmus; DA, ductus arteriosus; RAA with ALSA and left DA, right aortic arch with aberrant left subclavian artery and left ductus arteriosus; DAA, double aortic arch; RAA with mirror-image branching and RLDA, RAA with mirror-image branching and retroesophageal left ductus arteriosus.

**Figure 5:**
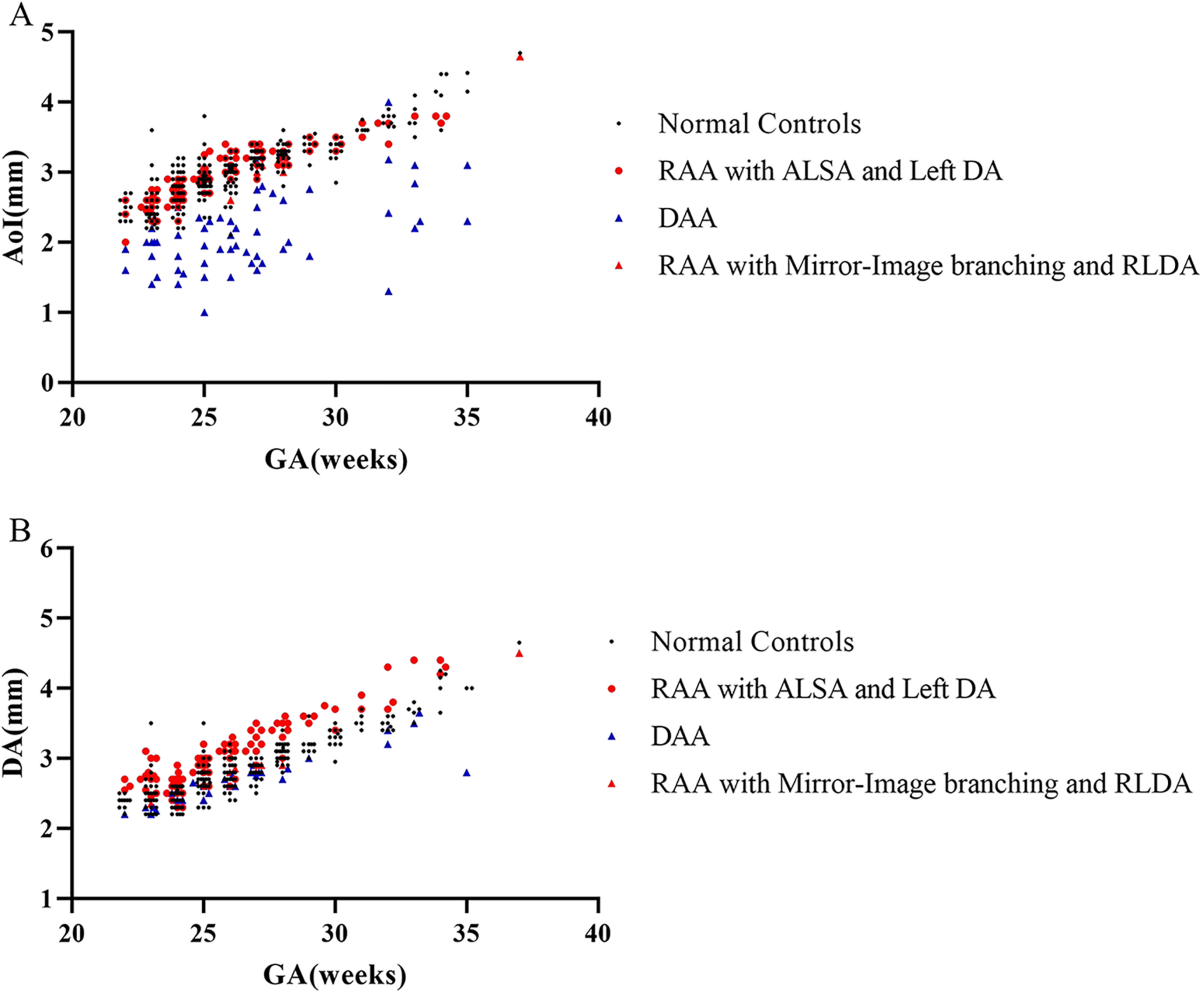
(**A,B**) Distribution of vascular diameters against gestational age measured by fetal MRI. Individual measurements of the diameters of AoI and DA in 130 CVR fetuses and 260 normal controls fetuses were plotted. (**A**) Scatter plot showed the relationships between diameters of AoI and GA in normal control group and three subgroups of CVR; (**B**) scatter plot showed relationships between diameters of DA and GA in normal control group and three subgroups of CVR. AoI, aortic arch isthmus; DA, ductus arteriosus; GA, gestation age; RAA with ALSA and left DA, right aortic arch with aberrant left subclavian artery and left ductus arteriosus; DAA, double aortic arch; RAA with mirror-image branching and RLDA, RAA with mirror-image branching and retroesophageal left ductus arteriosus.

In the normal control group fetuses, the mean diameter of AoI was 3.01 ± 0.46 mm, and the mean diameter of DA was 2.83 ± 0.43 mm. In three subgroups of CVR fetuses, the mean diameter of AoI was 3.01 ± 0.40 mm (RAA with ALSA and left DA), 2.15 ± 0.56 mm (DAA), 2.95 mm (2.68–3.08 mm) (RAA with mirror-image branching and RLDA) respectively; the mean diameter of DA was 3.06 ± 0.48 mm (RAA with ALSA and left DA), 2.71 ± 0.37 mm (DAA), 2.83 mm (2.50–2.90 mm) (RAA with mirror-image branching and RLDA) respectively. The diameters of AoI and DA (mean, SD, minimum, maximum, median, and 25th and 75th percentile) for each group were shown in Table 2. Compared with those of the control group, the AoI diameter of fetuses with DAA were decreased (*p* < 0.001), while the DA diameter of fetuses with RAA with ALSA and left DA was increased (*p* < 0.001). However, the AoI diameter of other two CVR subgroups (RAA with ALSA and left DA, RAA mirror-image branching and RLDA) was not statistically different from those of the control group (*p* = 0.924 and *p* = 0.738, respectively); and the DA diameter of other two CVR subgroups (DAA, RAA with mirror-image branching and RLDA) was also not statistically different from those of the control group (*p* = 0.133 and *p* = 0.900, respectively). Box-graphs showed the differences in AoI and DA diameters between the normal control group and the three subgroups of CVR, as shown in Figures 4A,B. The diameters of AoI and DA were positively correlated with GA in the normal control group (AoI: *r* = 0.886, *p* < 0.001; DA: *r* = 0.923, *p* < 0.001); The diameters of AoI and DA were also positively correlated with GA in RAA with ALSA and left DA subgroup (AoI: *r* = 0.896, *p* < 0.001; DA: *r* = 0.915, *p* < 0.001) and RAA with mirror-image branching and RLDA subgroup (AoI: *r* = 0.890, *p* = 0.003; DA: *r* = 0.909, *p* = 0.002); The diameters of DA were positively associated with GA in DAA subgroup (*r* = 0.884, *p* < 0.001), however, there was no linear tendency between the diameters of AoI and GA in the DAA subgroup (*r* = 0.336, *p* = 0.074). Scatter plots of AoI and DA diameters against GA were shown in Figures 5A,B.

**Table 2:** The tabular summaries of mean, SD, minimum, maximum, median, and 25th and 75th percentile from the base data of AoI and DA diameter (mm) respectively.

	Min		25th		50th		75th		Max		Mean		SD	
Groups	AoI	DA	AoI	DA	AoI	DA	AoI	DA	AoI	DA	AoI	DA	AoI	DA
Normal Controls	2.10	2.20	2.70	2.50	3.00	2.80	3.30	3.10	4.70	4.65	3.01	2.83	0.46	0.43
RAA with ALSA and Left DA	2.00	2.30	2.70	2.70	3.00	3.00	3.30	3.40	4.00	4.40	3.01	3.06	0.40	0.48
DAA	1.00	2.20	1.78	2.40	2.00	2.70	2.44	2.83	3.40	3.65	2.15	2.71	0.56	0.37
RAA with Mirror-Image branching and RLDA	2.45	2.35	2.68	2.50	2.95	2.83	3.08	2.90	4.65	4.50	3.08	3.05	0.67	0.97

AoI, aortic arch isthmus; DA, ductus arteriosus; Min, minimum; Max, maximum; SD, standard deviation; RAA with ALSA and left DA, right aortic arch with aberrant left subclavian artery and left ductus arteriosus; DAA, double aortic arch; RAA with mirror-image branching and RLDA, RAA with mirror-image branching and retroesophageal left ductus arteriosus.

In 130 fetuses with CVR, 13 cases were associated with other CHDs (especially ventricular septal defect, rather than complex heart disease), and 14 cases were with extracardiac malformation 16 cases were associated with airway compression with GA ranging from 25 to 30 weeks (mean, 27.5 weeks). The median tracheal diameter in fetuses with tracheal compression was 1.95 mm (1.80–2.20 mm). In normal fetuses with the matched gestational age, the median tracheal diameter was 2.94 mm (2.63–3.35 mm) (10). Compared with the normal tracheal diameter, the tracheal diameter in fetuses with tracheal compression was smaller (*p* < 0.0001). The associated CHD, extracardiac malformation, and tracheal compression are shown in Table 3.

**Table 3:** The tabular summaries of associated CHDs, extracardiac malformation, and airway compression.

Associated abnormalities	RAA with ALSA and Left DA	DAA	RAA with Mirror-image branching and RLDA
CHD			
VSD	*N* = 7	None	*N* = 1
TOF	*N* = 2	*N* = 1	None
Tricuspid regurgitation	*N* = 2	None	None
Extracardiac malformation			
PLSVC	*N* = 9	*N* = 2	None
ASLBV	*N* = 3	None	None
Airway compression	*N* = 15	None	*N* = 1

Three cases of RAA with ALSA and Left DA with duplicate counts, due to one suffering both tricuspid regurgitation and PLSVC simultaneously and two suffering both VSD and PLSVC simultaneously.

CHD, congenital heart disease; VSD, ventricular septal defect; TOF, tetralogy of Fallot; PLSVC, persistent left superior vena cava; ASLBV, abnormal subaortic left brachiocephalic vein; RAA with ALSA and left DA, right aortic arch with aberrant left subclavian artery and left ductus arteriosus; DAA, double aortic arch; RAA with mirror-image branching and RLDA, RAA with mirror-image branching and retroesophageal left ductus arteriosus.

Intra-observer (XZ) reproducibility for the diameters of AoI and DA and trachea was good (ICC = 0.966, 95% CI: 0.930–0.964; ICC = 0.968, 95% CI: 0.934–0.985; ICC = 0.929, 95% CI: 0.808–0.975); The inter-observer (S-ZD and MZ) reproducibility for the diameters of AoI and DA and trachea was acceptable (ICC = 0.925, 95% CI: 0.849–0.963; ICC = 0.903, 95% CI: 0.807–0.953; ICC = 0.901, 95% CI: 0.838–0.964).

## 4. Discussion

Although the prenatal identification of AA anomalies has significantly increased in the three-vessel view of echocardiography (11, 12), insufficient attention has been paid to the precise anatomy of the AA, DA, and brachiocephalic vessel branching pattern concerning the trachea. This study has demonstrated the value of fetal MRI in accurately distinguishing and quantifying various anatomical variants of AA and its branches.

Normal degeneration occurs when left AA is associated with the presence of left DA and common branching patterns (the right innominate artery, left common carotid artery and left subclavian artery). During embryonic development, abnormal degeneration of primitive paired AA leads to variations in AA positions and branching patterns (13). Fetal MRI can be a useful tool to provide excellent images of AA abnormalities and related anatomic structures (14). The most common fetal AA anomaly is an RAA (15), and the most common variation of RAA is RAA with ALSA and left DA (16). Our study also showed the most common variant of CVR was the RAA with ALSA and left DA, followed by DAA. It is necessary to accurately measure the diameters of the left and right aortic arches to determine the subtypes of DAA. In 29 confirmed cases of DAA in this study, the majority (66%) were right dominant arch subtypes. It is worth noting that when the left AA is small or atretic, just only a fibrous cord, DAA may be overlooked on prenatal MRI and misdiagnosed as RAA with mirror-image branching (17, 18). This misdiagnosis may result in ignoring postnatal tracheoesophageal compression. Most RAAs with mirror-image branching don’t form a vascular ring, however, RAA with mirror-image branching and RLDA is a rare form of CVR (19). The position of the DA and course of the left subclavian artery should be noted to determine whether there are vascular rings and possible types of variants.

Ultrasound is the first choice of method for measurement of fetal diameter of AoI, DA. There were some published studies on diameters of aortic arch and DA measured using ultrasound (20, 21). However, ultrasound has some limitations, including oligohydramnios, large gestational week, unfavorable fetal lie, and maternal obesity. MRI is not affected by above conditions, which particularly impair sonographic visualization of the fetal heart and provides a more detailed insight into vascular ring configuration (6). MRI can provide a large field of view of fetal CVR with excellent soft tissue resolution, and clearly display the airway morphology (6). Prenatal MRI measurements of fetal AA and DA diameters can accurately evaluate the variations of fetal AA. This study has shown that in RAA with ALSA and Left DA subgroup, the fetal DA diameter was significantly wider, which may be attributed to increased blood flow through DA in these cases. The increased blood flow through DA was due to the fact that DA not only transported blood flow from the pulmonary artery to the descending aorta but also undertook the blood supply of left subclavian artery. We also found a decrease in the diameter of AoI in the DAA subgroup due to the fact that the blood flow through the left and right AoIs was diverted and then decreased. However, in this study, there were no differences in AoI and DA diameters between fetuses with RAA with Mirror-Image branching and RLDA and fetuses in normal control group. Furthermore, it was found that the AoI and DA diameters were positively correlated with GA; The diameter of DA in the DAA subgroup is also positively correlated with GA in the normal control group, RAA with ALSA and left DA subgroup, and RAA with mirror branch and RLDA subgroup; and the diameters of DA were also positively associated with GA in DAA subgroup. These findings reflected the linear growth of fetal arteries with GA. However, there was no linear tendency between the diameters of AoI and GA in the DAA subgroup, possibly because the blood flow through the left and right AoIs was diverted and reduced unevenly. These differences in AoI diameter will help to identify at-risk fetuses, highlighting the need for continuous monitoring by prenatal imaging throughout pregnancy.

AA anomaly can be associated with other types of CHD, and the reported incidence of associated CHD varied from 10% to nearly 70% (11). The prognosis of fetuses with AA anomaly was mainly associated with the presence of associated defects, mainly CHD and complexity of CHD (22). In RAA cases, fetuses with vascular ring are less prone to CHD than fetuses with non-vascular ring (22). In a published study, the incidence of associated CHD in RAA with CVR was 16.2% (6/37, all CHDs were septal defects) (3). Our study has shown that the incidence of associated CHD was 10.0% (13/130), the most common CHD was also ventricular septal defect (6.2%, 8/130) and the most common associated extracardiac anomaly was persistent left superior vena cava (8.5%, 11/130).

Fetal MRI can show the location, extent, and severity of tracheal compression. CVR may cause severe tracheal stenosis and respiratory failure in infants, in the case, emergency surgery is required after birth (23). Prenatal detection of fetal tracheal compression can indicate a high possibility of postnatal symptoms (24). Prenatal recognition of severe tracheal compression is helpful for surgical intervention at the right time and monitoring the development of tracheal cartilage.

## Limitation

In this study, the limitation is that the sample size of RAA with Mirror-Image branching and RLDA was small. In the future, large sample prospective studies are needed to verify the effectiveness of these measured parameters, as well as fetal AA and branching pattern variants, in predicting postpartum outcomes.

## Conclusions

The altered diameters of AoI and DA can be detected and measured in CVR fetuses in transverse aortic arch view using fetal cardiovascular MRI. Fetal CVR can occur alone or with intracardiac and extracardiac malformation. Fetal CVR can be associated with prenatal airway compression.

## Data Availability

The raw data supporting the conclusions of this article will be made available by the authors, without undue reservation.
